# Validation of fluorescent dust marking of *Culicoides* biting midges and the design of a self-marking technique

**DOI:** 10.1186/s13071-015-0657-0

**Published:** 2015-01-27

**Authors:** Georgette Kluiters, Kristina Hunter, Matthew Baylis

**Affiliations:** Liverpool University Climate and Infectious Diseases of Animals (LUCINDA) Group, Institute of Infection and Global Health, University of Liverpool, Leahurst Campus, Neston, Cheshire CH64 7TE UK; National Institute for Health Research, Health Protection Research Unit in Emerging and Zoonotic Infections, University of Liverpool, Leahurst Campus, Neston, Cheshire CH64 7TE UK

**Keywords:** *Culicoides*, Mark-release-recapture, MRR, Dispersal, Fluorescent, Dust, Marking, Self-marking

## Abstract

**Background:**

Investigation of insect flight patterns frequently involves the use of dispersal studies. A common method for studying insect dispersal is mark-release-recapture (MRR) techniques using wild-caught insects in their natural environment; however, this requires a suitable marker. At present, no studies have been performed to identify markers that are suitable for use in midges within the Obsoletus Group, and visible by eye or down a light microscope.

**Methods:**

A series of 11 experiments were undertaken to determine the effectiveness of three colours of Brilliant General Purpose (BGP) fluorescent dusts in marking *Culicoides* midges. Three areas were focused on: 1) dust properties, 2) the effect on *Culicoides*, and 3) dust application in the field.

**Results:**

All three dusts were insoluble in water, 10% washing-up liquid and 70% ethanol. They were visible down a microscope, with and without the use of a black light, and two were highly visible without the need for a microscope. The dusts remained adherent to the marked *Culicoides* for the duration of the experiments, did not transfer between marked and unmarked individuals or the environment, and remained adherent when the *Culicoides* were stored in an ethanol or water-based solution. The dusts had no effect on the mortality rate of the insects over the 48 hrs of the experiment. There were no significant differences between the recorded behaviours undertaken by undusted control *Culicoides* and the BGP fluorescent dusted *Culicoides*. Field-based marking of *Culicoides* can be achieved using a ‘self-marking’ technique, whereby the trapping vessel is pre-dusted with fluorescent dust prior to trapping the individuals to be marked.

**Conclusions:**

This is the first study to identify BGP fluorescent dusts as markers for use with Obsoletus Group *Culicoides*. BGP fluorescent dusts provide a quick and effective method of marking and identifying *Culicoides* for both field and laboratory studies. The self-marking technique minimises the time needed to handle specimens prior to release.

## Background

*Culicoides* flight behaviour is believed to drive the dispersal of midge-borne diseases, such as bluetongue (BT), from farm-to-farm; and modelling studies suggest that BT outbreaks cannot occur in the absence of local spread by midges [1]. *Culicoides* flight behaviour is therefore a critical aspect of BT epidemiology, but it remains poorly understood [2].

Investigation of insect flight patterns frequently involves the use of dispersal studies [3-5]. Different approaches have been attempted to study the dispersal of *Culicoides* spp. midges: long-distance dispersal studies have utilised evidence from disease outbreaks [6-10], whereas short-distance studies have involved direct capture of adult midges near breeding sites. An optimal approach would be a mark-release-recapture study (MRR) using wild-caught midges in their natural environment, however this is only possible with the availability of a suitable marking agent. Any selected marker must not have a detrimental effect on the survival or behaviour of marked specimens or the environment, but must also remain adherent under adverse conditions, be highly visible and allow differentiation between marked and unmarked individuals [11].

The Palaearctic BT vectors, members of the Obsoletus Group, are highly abundant throughout northern Europe [12], yet their dispersal ability has not been determined. Until now, dispersal studies have only been successfully undertaken on *C. mississippiensis* [4], *C. mohave* [13] and *C. variipennis* [14]*,* as well as the Pulicaris Group in Denmark [15]. As such, investigation of marking agents has mainly been undertaken on these species. A recent study in Denmark investigated the use of fluorescein isothiocyanate (FITC) to mark *Culicoides* and successfully employed this marker in a MRR experiment [15]. The marker has a number of drawbacks, however, as it requires a plate scanner and associated software for the detection of FITC on individual specimens, is removed from *Culicoides* with the addition of ethanol*,* and is light-sensitive so could fade over time on *Culicoides* released in the field. The impact of FITC on the survival rate of *Culicoides* was also not tested, and spurious results may occur if *Culicoides* were to become contaminated with other autofluorescent matter, such as some types of pollen, in the field.

Although the use of micronized fluorescent dusts have produced good results in a laboratory setting [4], and also proved successful in field trials [4,13,14], a number of problems still remain. Firstly, the use of easily identifiable fluorescent dusts that can be seen by eye have not been tested on Obsoletus Group members, now identified as major vectors of viruses in Europe. Secondly, the manufacturers of such dusts used in previous studies no longer exist.

The aim of this study was therefore to determine whether Obsoletus Group members could be marked effectively using micronized fluorescent dusts under both laboratory and field settings. Specific objectives included testing the hypothesis that the dusts have no adverse effects on *Culicoides* behaviour, life-span or flight compared to unmarked controls. The ability to detect marked individuals trapped in both water and ethanol was investigated, as well as whether any dust transfer occurs from marked individuals to other *Culicoides* or the environment. As no self-marking method currently exists for marking *Culicoides* in the field the final aim of this set of experiments was to explore the possibility of a self-marking technique. This paper is one of a pair of companion papers, the second of which uses the fluorescent dusts and the self-marking method devised in this paper in a MRR study on Palaearctic *Culicoides* [16].

## Methods

### Selection of fluorescent dusts

Brilliant General Purpose (BGP) Fluorescent Pigments manufactured by Brilliant Group (San Francisco, USA) were selected as the fluorescent dusts to be trialled due to their small particle size (3–5 microns), wide range of colours which would be useful for repetitions of mark-release-recapture (MRR) experiments, non-toxic nature, and availability. The BGP series of fluorescent pigments are principally used in the coloration of paints, coatings, inks and plastics. Three colours were selected to be trialled as marking agents – pink (BGP-PK111), green (BGP-GR118) and yellow (BGP-YE117).

### Marking method

**Figure 1:**
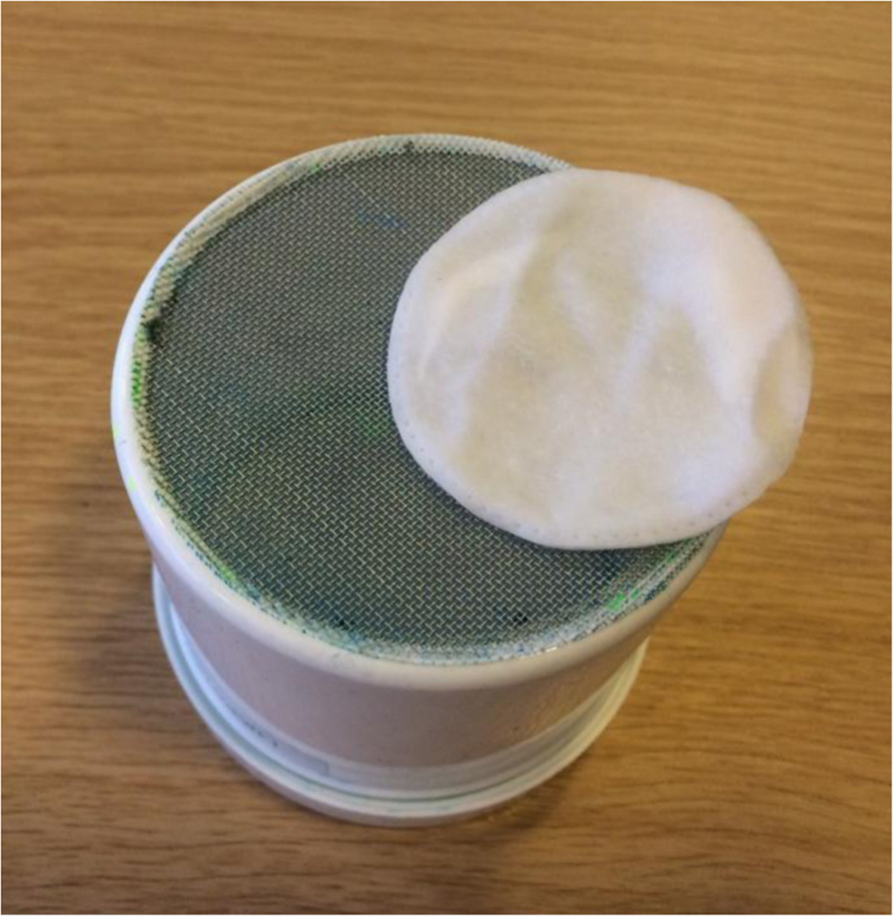
**The maintenance of** ***Culicoides*** **in plastic trapping containers.** Cotton wool embedded with a 10% sucrose solution was placed on top of the gauze lids for sustenance. The same trapping pots were used for self-marking *Culicoides* with the fluorescent dusts in the field.

In July 2010 the use of Brilliant General Pigment (BGP, Brilliant Group, Inc., San Francisco, USA) micronized fluorescent dusts in marking *Culicoides* for dispersal studies was investigated using a series of 11 laboratory studies falling under three areas of interest; 1) dust properties; 2) effect of dust on *Culicoides*; and 3) dust application. *Culicoides* were collected at the University of Liverpool’s Leahurst Campus using an Onderstepoort-type down-draught black light trap placed at 2 m in height, and were stored in plastic trapping containers with a gauze bottom with a 10% sucrose solution embedded in cotton wool for sustenance (Figure 1). During the following experiments, midges were incapacitated on a cold plate set to −15°C for 15 seconds to allow for the separation of members of the Obsoletus Group according to their wing patterns. *Culicoides* were killed following transfer to a −80°C freezer for 20 min.

#### Investigation of dust properties

The solubility of the dusts was determined in three test solutions of 100 ml of water, a 10% detergent solution and 70% ethanol prepared in triplicate and held at 10°C, 20°C and 30°C. Exactly 1 g of each dust (green, pink and yellow) was added individually to the test solutions, and the solutions were agitated using a magnetic stirrer (Stuart Heat-stir SB162, Bibby Scientific Limited, UK) for 30 minutes. Dust solubility was assessed via filtration of beaker contents using 5 μm filtration-paper. The tests were repeated using 0.01 g of each coloured dust.

Dust visibility was assessed on 30 killed *Culicoides* by dusting them with 0.01 g of each dust while stored in a 15 ml tube, and then inspecting them using a stereomicroscope under both natural and black light, before live-dusted *Culicoides* were inspected from the experiments that followed.

Dust adherence was initially tested on 30 killed *Culicoides* which were added to 0.01 g of the marker in a 15 ml tube, agitated with an electronic shaker and inspected for dust coverage, before being stored at 10°C and rechecked at 24 and 48 hrs. The experiment was repeated, with marked midges added to 10 ml test solutions of water, 10% detergent solution or 70% ethanol, before being held at 10°C, 20°C or 30°C for 30 min, after which time the *Culicoides* were then individually removed and examined for dust adherence. These specimens were further stored and rechecked at 24 and 48 hrs and the solutions fluoresced under a blacklight to check for the present of fluorescent dust.

To determine dust transfer to the environment, 30 live *Culicoides* were transferred to a 15 ml tube containing 0.01 g of fluorescent dust and the tube gently rolled to mark the insects. Marked *Culicoides* were transferred into gauze-ended trapping beakers with paper towel-lined bases for 48 hrs before being killed and removed, and the paper towel and container surfaces inspected for dust transfer using a black light.

#### Effects of dust on culicoides

Dust toxicity was assessed by transferring 30 live-marked (using the tube rolling method above) and 30 unmarked *Culicoides* into gauze-ended trapping beakers and the mortality rate of both groups was recorded every 30 min for 4 hours, with *Culicoides* then rechecked at 24, 48 and 72 hrs. The effect of the dusts on *Culicoides* survival was determined by survival analysis, using the log-rank test, and presented as a Kaplan Meier graph.

Scan sampling was used to determine the impact of dusts on behaviour of 30 live-marked individuals against 30 unmarked control *Culicoides* held in separate containers. The proportion of midges exhibiting six listed behaviours (flying, climbing, walking, feeding, cleaning and resting) at 5 min intervals for the first hour, every 15 min for the second hour, and 30 min for the third hour was compared between the groups. The effect of the dusts on the behaviour *Culicoides* was assessed using chi-square analyses.

The transfer of fluorescent dust between *Culicoides* was assessed by placing 30 live-marked and 30 unmarked *Culicoides* into a gauze-ended trapping beaker for 24 hrs before being killed and inspected under a microscope.

#### Dust application

Three approaches to marking were compared to determine ease of application, evenness of coverage, mortality, injury and waste residue. The methods involved using a fine brush to sieve dust through the gauze surface of a trapping pot containing *Culicoides*, using a syringe to inject dust into a vacuum flask containing midges and, pre-dusting the inside surface of trapping pots [Figure 1 shows a trapping pot] to create a fine layer of dust before *Culicoides* were placed in the pots and allowed to walk independently over the surfaces.

Due to the success of the pre-dusted trapping pot method, pots were further pre-dusted with 0.1 g, 0.25 g, 0.5 g, 0.75 g or 1 g of dust and attached to an OVI trap at the University of Liverpool’s Leahurst Campus where the trap was run overnight and the proportion of marked midges determined the following day. The mortality rate of the *Culicoides* collected overnight was determined using chi-square analysis.

## Results

### Investigation of dust properties

Both 0.01 g and 1 g of the three fluorescent dusts were insoluble in water, 10% washing-up liquid solution and ethanol at 10, 20 and 30°C. The dusts were visible, both directly and under a stereomicroscope in natural or black-light conditions, with no variation in visibility detected. They were visible on all surfaces dusted, with no difference in visibility identified.

The three dust markers adhered to all midges for the duration of the experiment (48 hours). BGP yellow and green dusts demonstrated greatest attachment to the wings, wing base and legs, while BGP Brilliant Pink adhered to the thorax. Five of the killed C*ulicoides* marked by agitating the pre-dusted tubes on an electronic shaker showed damage to their wings and antennae, while *Culicoides* marked via rotation of the tube exhibited no such damage.

Following storage of the killed and marked *Culicoides* in water, 10% detergent solution, and 70% ethanol for 48 hrs, the dusts remained adherent on the individuals and no extraneous particles were found either on the container or liquid they were stored in. The results did not vary with temperature. No transfer occurred with any dust from marked midges to any of the test surfaces over the 48 hours of the experiment.

### Effect of dust on Culicoides

**Figure 2:**
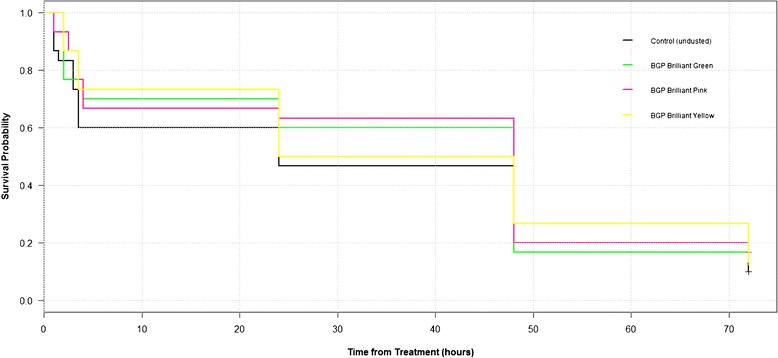
**Kaplan Meier survival curves for** ***Culicoides*** **dusted with BGP green, pink or yellow fluorescent dust and un-dusted control** ***Culicoides*** **.**

There were no differences in the survival between the un-dusted control *Culicoides* and those dusted with pink, yellow or green fluorescent dust (log-rank test, *P =* 0.914). Kaplan Meier survival curves for the dusts can be seen in Figure 2.

**Figure 3:**
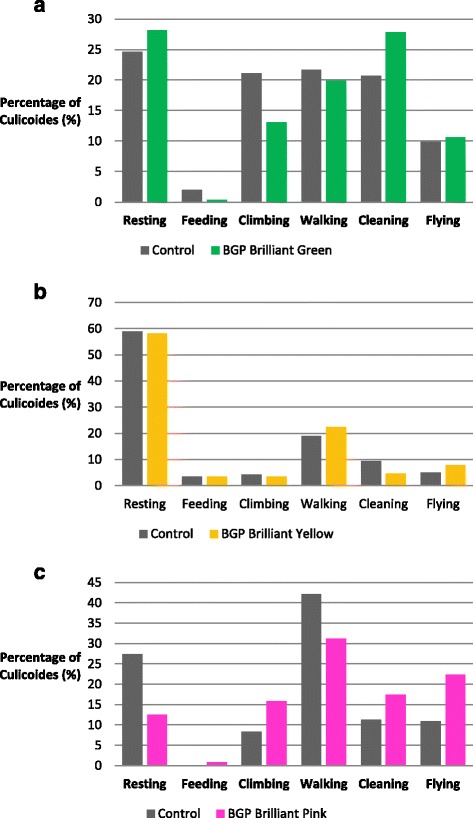
**Percentage of** ***Culicoides*** **exhibiting six observed behaviours when dusted with fluorescent dusts in comparison to un-dusted control** ***Culicoides;*** where **a)** uses Brilliant General Purpose (BGP) Green fluorescent dust; **b)** BGP Yellow fluorescent dust; and **c)** BGP Pink fluorescent dust.

Overall there were no significant differences between the behaviours undertaken between the undusted control *Culicoides* and the BGP fluorescent dusted *Culicoides* (chi-square, *P* = 0.922). Differences were observed between the three control populations of unmarked *Culicoides* (chi-square, *P ≤* 0.001). The percentage of *Culicoides* exhibiting the listed behaviours during the experiment can be seen in Figure 3.

No dust transfer occurred between dust-marked *Culicoides* and unmarked controls, with 30 marked *Culicoides* and 30 unmarked control *Culicoides* identified using a stereomicroscope at the end of the experiment.

### Dust application

Of the three dust application methods trialled in the laboratory (fine brush technique, syringe injection, pre-dusted containers), the syringe-based method proved the most difficult in terms of accurately measuring the required amount of dust for application, also leading to wastage with dust remaining in the syringe. This method also led to a limited number of individuals being marked within the time limit of the experiment (14 individuals out of 30). Four individuals also exhibited injury to their wings when this method was employed.

Although the required amount of dust was easy to measure when using the fine brush technique, it was difficult to obtain an even coverage of individuals through the mesh gauze of the trapping pot, with some individuals receiving a higher coverage of dust than others (although all 30 midges were marked). The marking method worked best when the pot itself was already pre-dusted with fluorescent dust prior to the *Culicoides* being added (all 30 *Culicoides* marked). There was no mortality using any method.

The number of *Culicoides* trapped during each replicate of the field-marking experiment can be seen in Table 1. The total *Culicoides* trapped during the 5 consecutive trapping nights was 880. Of these, 100% of individuals were marked each night, irrespective of the weight of dust used to pre-dust the container. The mortality rate of *Culicoides* did not vary significantly between different dust weights (chi-square, *P* = 0.1).

**Table 1:** **The number of** ***Culicoides*** **trapped, marked and dead when caught overnight in trapping containers pre-dusted with differing quantities of fluorescent dusts**

**Weight of dust (g)**	**Number of** ***culicoides*** **trapped**	**Number of** ***culicoides*** **marked**	**Number of dead** ***culicoides***
**0.1**	134	134	0
**0.25**	217	217	2
**0.5**	203	203	0
**0.75**	167	167	2
**1.0**	159	159	4

## Discussion

This study successfully identifies a fluorescent dust marker and self-marking technique for members of the Obsoletus Group of *Culicoides*, the vectors of bluetongue virus in northern Europe. To our knowledge, there have been no studies published on the use of fluorescent dusts for marking *Culicoides* since the 1980s [4,13,14], and the fluorescent dusts employed in these early studies are no longer available. This technique should be useful in studying the dispersal behaviour of members of the Obsoletus Group under field conditions.

The dust properties tested indicate that the fluorescent dusts could be successfully used in mark-release-recapture (MRR) experiments. As all dusts remained insoluble in water, 10% detergent solution, and 70% ethanol this would allow trapping during field investigations to be undertaken using standard trapping solutions of water with a drop of washing-up liquid in order to break the surface tension. It would also allow storage of insects in 70% ethanol solutions following collection. Similarly, the adherence experiments suggested that not only are the dusts insoluble in these test solutions, but they also remain adhered to the *Culicoides* themselves while in solution. As adherence to *Culicoides*, when stored in ethanol, was only evaluated for 48 hrs in this experiment, more work would need to be undertaken to determine if long-term storage in ethanol would affect the adherence of the dusts.

Dust visibility was not reduced after the marked *Culicoides* were stored in solution. The use of a fluorescent light was not always required to detect marked individuals, as the pink and green fluorescent dusts were easily identifiable on the *Culicoides* without being fluoresced. The use of these dusts therefore provides a quickly identifiable marker, eliminating the need for more complex and time-consuming detection methods.

Transfer of fluorescent dust to the environment in which the marked *Culicoides* were held was not observed over the 24 hour period they were examined for. As it is likely that any superfluous dust would have been removed by the *Culicoides* rapidly, it is unlikely that dust transfer would occur at any time after this 24 hour period. Other researchers have incorporated dusts with gum arabic, so that particles could not easily be removed with preening and wing movements [17,18], but that was not necessary here.

While visibility and adherence of the marker is of utmost importance, any selected marker must also not have a detrimental effect on the survival or behaviour of marked specimens [11]. Although an increase in mortality of marked *Culicoides* was observed over time, a similar increase was also observed in the unmarked controls.

Of the markers tested, there were no significant differences between the behaviours of the un-marked controls and the marked *Culicoides*, highlighting that application of the dusts as a marking agent in the field would not affect the results obtained by MRR experiments. Differences were observed, however, between the populations of control *Culicoides* used for each pair of experiments and this is likely to be due to the pairs of experiments being undertaken on different days. Although environmental conditions, such as temperature, were kept stable within the laboratory, the differences between days may be linked to different trapping conditions on each night (e.g. temperature or rainfall), the length of time *Culicoides* were held in the live-trapping containers prior to the start of the behaviour experiment, or the number of *Culicoides* trapped in the live-trapping pot over night.

Although many important aspects of a marking agent were tested in this study, the laboratory environment within which the experiments were undertaken is unlikely to be identical to the conditions *Culicoides* would encounter in the field. We did not assess the impact of rain or wind on the adherence of the dusts to *Culicoides*, or their subsequent transfer to the environment. Similarly, the duration of these experiments was limited due to the difficulties encountered with keeping wild-caught Obsoletus Group members alive in a laboratory environment. Although laboratory colonies of other *Culicoides* species are available, the behaviours of these individuals may be markedly different from those of their wild counterparts and as a colony of Obsoletus Group members has not been established, wild-caught individuals were employed here.

Following the series of laboratory experiments on the dusts a small field-trial of a self-marking method was successfully employed. The field-study highlighted the ease of use of the pre-dusted trapping pot method in self-marking *Culicoides* for MRR studies, eliminating the need to manually apply dust to the insects the following morning. As no difference in mortality rates or dust coverage of *Culicoides* occurred when different quantities of dusts were applied to the pots, we recommend that a sufficient quantity of dust is applied so as to fully cover the sides and gauze bottom of the trapping pot, with this quantity varying depending on the size of trapping vessel used.

Relatively small numbers of *Culicoides* were trapped during the field-trial so it would be useful to replicate the trial in an area where larger numbers of insects are likely to be trapped to determine whether dust coverage remains at 100% with large numbers of insects. A MRR study was undertaken in a companion paper using the BGP fluorescent dusts and the self-marking method described here [16].

## Conclusions

This is the first study to identify BGP fluorescent dusts as markers for use with Obsoletus Group *Culicoides*. BGP fluorescent dusts are a suitable marking agent for *Culicoides* midges as they do not influence either survival or flight behavior of Obsoletus Group members in the laboratory. Marked midges remained distinguishable for their entire lifetime during the experiment; dusts did not transfer from marked to unmarked individuals or the environment; the mortality rate of marked midges did not differ from controls under laboratory conditions; and, importantly for trapping and storing *Culicoides*, the dust did not dissolve or wash off in either ethanol or water. Pre-dusting trapping pots with BGP fluorescent dusts prior to trapping provides a fast and reliable method for self-marking *Culicoides* in the field and should prove useful for MRR studies.

## Abbreviations

BGP: Brilliant General Purpose
MRR: Mark-release-recapture

## References

[1] Turner J, Bowers RG, Baylis M (2012). Modelling bluetongue virus transmission between farms using animal and vector movements. Sci Rep.

[2] Carpenter S, Wilson A, Mellor PS (2009). *Culicoides* and the emergence of bluetongue virus in northern Europe. Trends Microbiol.

[3] Danthanarayana W (1986). Insect flight. Dispersal and migration. Entomol Exp Appl.

[4] Lillie T, Kline D, Hall D (1985). The dispersal of *Culicoides mississippiensis* (Diptera: Ceratopogonidae) in a salt marsh near Yankeetown, Florida. J Am Mosq Control Assoc.

[5] Valerio L, Facchinelli L, Ramsey JM, Bond JG, Scott TW (2012). Dispersal of male *Aedes aegypti* in a coastal village in southern Mexico. Am J Trop Med Hyg.

[6] Burgin LE, Gloster J, Sanders C, Mellor PS, Gubbins S, Carpenter S (2013). Investigating incursions of bluetongue virus using a model of long-distance *Culicoides* biting midge dispersal. Transbound Emerg Dis.

[7] Ducheyne E, De Decken R, Becu S, Codina B, Nomikou K, Mangana-Vougiaki O (2007). Quantifying the wind dispersal of *Culicoides* species in Greece and Bulgaria. Geospat Health.

[8] Eagles D, Deveson T, Walker PJ, Zalucki MP, Durr P (2012). Evaluation of long-distance dispersal of *Culicoides* midges into northern Australia using a migration model. Med Vet Ent.

[9] Hendrickx G, Gilbert M, Staubach C, Elbers A, Mintiens K, Gerbier G (2008). A wind density model to quantify the airborne spread of *Culicoides* species during north-western Europe bluetongue epidemic. Prev Vet Med.

[10] Sedda L, Brown HE, Purse BV, Burgin L, Gloster J, Rogers DJ (2012). A new algorithm quantifies the roles of wind and midge flight activity in the bluetongue epizootic in northwest Europe. Proc R Soc B, Biological Sciences.

[11] Hagler JR, Jackson CG (2001). Methods for marking insects: current techniques and future prospects. Annu RevEntomol.

[12] Kluiters G, Sugden D, Guis H, McIntyre KM, Labuschagne K, Vilar MJ (2013). Modelling the spatial distribution of *Culicoides* biting midges at the local scale. J App Ecol.

[13] Brenner R, Wargo M, Stains G, Mulla M (1984). The dispersal of *Culicoides mohave* (Diptera: Ceratopogonidae) in the desert of southern California. Mosq News.

[14] Lillie T, Marquardt W, Jones R (1981). The flight range of *Culicoides variipennis* (Diptera: Ceratopogonidae). Can Entomol.

[15] Kirkeby C, Bødker R, Stockmarr A, Lind P, Heegaard PMH (2013). Quantifying dispersal of European *Culicoides* (Diptera: Ceratopogonidae) vectors between farms using a novel mark-release-recapture technique. PLoS One.

[16] Kluiters G, Swales H, Baylis M. Local dispersal of Palaearctic *Culicoides* biting midges estimated by mark-release-recapture. Parasit Vectors*.* 2015;8:54. 10.1186/s13071-015-0658-z PMC4327803 25886488

[17] Sinsko MJ, Craig GB (1979). Dynamics of an isolated population of Aedes triseriatus (Diptera: Culicidae). I. Population size. J Med Entomol.

[18] Brust RA (1980). Dispersal behaviour of adult Aedes sticticus and Aedes vexans (Diptera: Culicidae) in Manitoba. Can Entomol.

